# The Role of Oncogenic Tyrosine Kinase NPM-ALK in Genomic Instability

**DOI:** 10.3390/cancers10030064

**Published:** 2018-03-05

**Authors:** Cosimo Lobello, Vasilis Bikos, Andrea Janikova, Sarka Pospisilova

**Affiliations:** 1 Central European Institute of Technology (CEITEC), Masaryk University, Kamenice 5, 62500 Brno, Czech Republic; cosimo.lobello@ceitec.muni.cz (C.L.); vasileios.bikos@ceitec.muni.cz (V.B.); 2 Department of Internal Medicine—Hematology and Oncology, University Hospital Brno, 62500 Brno, Czech Republic; janikova.andrea@fnbrno.cz

**Keywords:** NPM-ALK (nucleophosmin-anaplastic lymphoma kinase), ALK, anaplastic large cell lymphoma (ALCL), DNA damage response, genomic instability, DNA repair, p53, MutS protein homolog 2 (MSH2)

## Abstract

Genomic stability is crucial for cell life and transmitting genetic material is one of the primary tasks of the cell. The cell needs to be able to recognize any possible error and quickly repair it, and thus, cells have developed several mechanisms to detect DNA damage and promote repair during evolution. The DNA damage response (DDR) and DNA repair pathways ensure the control of possible errors that could impair the duplication of genetic information and introduce variants in the DNA. Endogenous and exogenous factors compromise genomic stability and cause dysregulation in the DDR and DNA repair pathways. Cancer cells often impair these mechanisms to overcome cellular barriers (cellular senescence and/or apoptosis), leading to malignancy. NPM (nucleophosmin)-ALK (anaplastic lymphoma kinase) is an oncogenic tyrosine kinase that is involved in the development of anaplastic large cell lymphoma (ALCL). NPM-ALK is known to be involved in the activation of proliferative and anti-apoptotic signaling pathways. New evidence reveals that NPM-ALK translocation also impairs the ability of cells to maintain the genomic stability through both DDR and DNA repair pathways. This review aims to highlight the role of the oncogenic tyrosine kinase NPM-ALK in the cell, and pointing to new possible therapeutic strategies.

## 1. Introduction

Cells have to robustly counteract against events that can cause DNA damage, and, be ready to recognize and repair any possible error in genetic material. They are subjected to a high degree of external stress daily, from sources such as irradiation, UV light, reactive oxygen species (ROS), tobacco smoke, or from the cellular environment, such as errors that are formed during DNA replication by DNA polymerases. Since there are a plethora of possible factors that can induce different types of damage in the cell’s genomic integrity, each error has a specific way to be recognized and treated. The genome-maintenance machinery is well organized: cells need to recognize the error by sensor proteins, communicate the problem using signal transducers, and then fix it through effectors [1]. There are two main mechanisms responsible for these essential tasks: DNA damage response and DNA repair pathways. Damage or mutations in these two pathways are often related to genomic instability and cancer development. A case in point, as has been shown, is that oncogenic tyrosine kinases, such as NPM (nucleophosmin)-ALK (anaplastic lymphoma kinase) (and also BCR (breakpoint cluster region)-ABL (abelson murine leukemia viral oncogene homolog 1), TEL (translocation ETS leukemia)-ABL, TEL-JAK2 (janus kinase 2)) induce DNA damage, and directly impair the normal function of proteins that are involved in these pathways [2]. For instance, BCR-ABL was found to be directly involved in the regulation of DNA repair decreasing the expression of DNA-dependent protein kinase, catalytic subunit (DNA-PKcs) in patients with chronic myeloid leukemia (CML) [3]. DNA-PKcs is a kinase protein that is involved in the non-homologous end joining (NHEJ) pathway of DNA repair. Down-regulation of DNA-PKcs by BCR-ABL is related to DNA repair deficiency and it plays a role in CML progression [4].

### The Oncogenic Tyrosine Kinase NPM-ALK

The translocation t(2;5) (p23;q35) was discovered for the first time in the late 1980s in anaplastic large cell lymphoma (ALCL) [5]. Subsequently, in 1994, the product of this translocation was identified as the fusion between the receptor anaplastic lymphoma kinase (ALK) and nucleophosmin (NPM1) loci, on chromosomes 2 and 5, respectively [6]. Since that time, many studies were focused on understanding of the role of NPM-ALK translocation. ALK is a receptor protein-tyrosine kinase and its aberration is observed in several human malignancies, such as ALCL, neuroblastoma, inflammatory myofibroblastic tumor, or non-small cell lung carcinoma (NSCLC) [7]. The translocation with NPM is the most common in ALCL, as it is present in about 80% of ALK-positive ALCL [8], although several other ALK partner genes are described, such as TFG, TPM3, ATIC [9,10,11]. NPM-ALK fusion protein is constitutively activated in ALCL, and after its dimerization it activates various signaling pathways, including phosphatidylinositol-3-kinase(PI3K)/AKT/mTOR, JAK/signal transducer and activator of transcription 3 (STAT3), RAS/ERK, and phospholipase C (PLCγ) [12,13,14]. Through the deregulated activation of these pathways, NPM-ALK induces cell-cycle progression, proliferation, cell survival and anti-apoptotic functions [15]. In this review, we focus on the involvement of NPM-ALK translocation in the tumor pathogenesis, by influencing pathways that are dedicated to the maintenance of genomic stability.

## 2. DNA Damage Response and Role of Tumor Suppressor p53

The cell evolved extremely powerful machinery to protect itself from damage—DNA damage response (DDR) pathways. Various types of damage can affect the cell: single point mutations, single-strand DNA breaks (SSBs) or double-strand DNA breaks (DSBs). The main actors in DNA damage response are two kinases: ATM (ataxia telangiectasia mutated) and ATR (ATM-Rad3-related) [16]. ATM and ATR are upstream molecules of the main DDR pathways dedicated to recognizing DSBs and SSBs, respectively [17]. Once the sensor proteins detect the DNA damage, these two kinases are recruited and trigger DNA damage response cascades. These cascades include (i) cellular checkpoints, able to stop cell cycle progression, (ii) the DNA repair pathway, to rectify possible mistake(s), and (iii) the apoptotic pathway, to induce cell death when the damage resolution is not possible. In recent decades, several studies clarified how these kinases are initially triggered, and identified a pool of proteins that directly bind the DNA in the broken sites. The Mre11-Rad50-Nbs1 (MRN) mediator complex identifies the DSB and recruits ATM to the broken DNA site [18,19]. Another complex mechanism is required for ATR recruitment; when the replication protein A (RPA) binds the ssDNA [20], it triggers the enrollment of ATR through ATR interacting protein (ATRIP) [21] and the activation of Rad9-Rad1-HUS1 and Rad17-RFC complexes [16,17], allowing for ATR to interact with the damage. ATM and ATR share downstream targets, including checkpoint kinase 1 and 2 (CHK1and CHK2) [22] and components of p53 pathway [23]. Indeed, ATM and ATR mediate the binding degradation of p53 with its negative regulators, murine double minute 2 (MDM2), or murine double minute 4 (MDM4) [24,25]. Though it is still not entirely clear how p53 is recruited, evidence suggests that DNA damage and genomic instability might induce the activation of p53 to accomplish tumor suppression [26]. In this scenario, the tumor suppressor p53 plays a central role in the cell’s fate. p53 is a transcription factor and a master regulator of the cell cycle. After a stressful event, such as DNA damage, the cell can go through either cell-cycle arrest or apoptosis, and p53 is a crucial protein for this decision [27]. In stress conditions, it receives the signal and induces the activation of a spectrum of cellular response involved in cell cycle arrest, DNA repair, autophagy, apoptosis and other activities according to the level of damage suffered by the cell. The transcription factor p53 has different domains: a N-terminal transactivation domain (TAD), a proline-rich domain (PRD), a core DNA binding domain (DBD), a tetramerization domain (4D), and a C-terminal regulatory domain (CTD) [28]. Somatic mutation or deletion of the *TP53* gene are the most common and well-documented mechanisms by which p53 activity is deregulated. Moreover, damage in the p53-regulator pathways, such as the overexpression of its negative regulators MDM2 or MDM4, but also epigenetic modification, miRNAs alteration or *TP53* splicing deregulation, can impair p53 activity [29]. The level of p53 is essential and is strictly controlled by the cell. Under normal conditions, p53 is negatively regulated by MDM2 or MDM4, which bind the TAD domain of p53, inducing the degradation of the protein by ubiquitination [30,31]. The balance between p53 and MDM2 is crucial for p53 activation. In fact, p53 activates MDM2 transcription, inducing negative feedback on its expression. This balance is altered by DNA damage that increases p53 levels and induces post-translational modification of MDM2. In this situation, MDM2 is not able to negatively regulate p53, allowing for the activation of p53 gene targets [32,33]. Activated p53 regulates the expression of a plethora of genes that are involved in multiple cellular functions, such as (i) cyclin dependent kinase inhibitor 1A (CDKN1A), by the transcription regulation of which it is able to halt the cell at the G1 phase, allowing to the cell to have sufficient time to repair the DNA damage and restore genomic stability, (ii) Bcl-2-binding component 3 (BBC3) and Bcl-2-associated X (BAX) in apoptosis or (iii) promyelocytic leukemia protein (PML) in cellular senescence [34]. Defects in ATM, ATR, and p53 have been described in B and T-cell lymphoma [35,36]. For instance, alterations in *TP53* and *ATM*, due to deletions or mutations, have been associated with poor prognosis and chemoresistance in chronic lymphocytic leukemia (CLL) and their detection has become clinically necessary [37,38,39]

### p53 in ALK-Positive Anaplastic Large Cell Lymphoma

**Figure 1:**
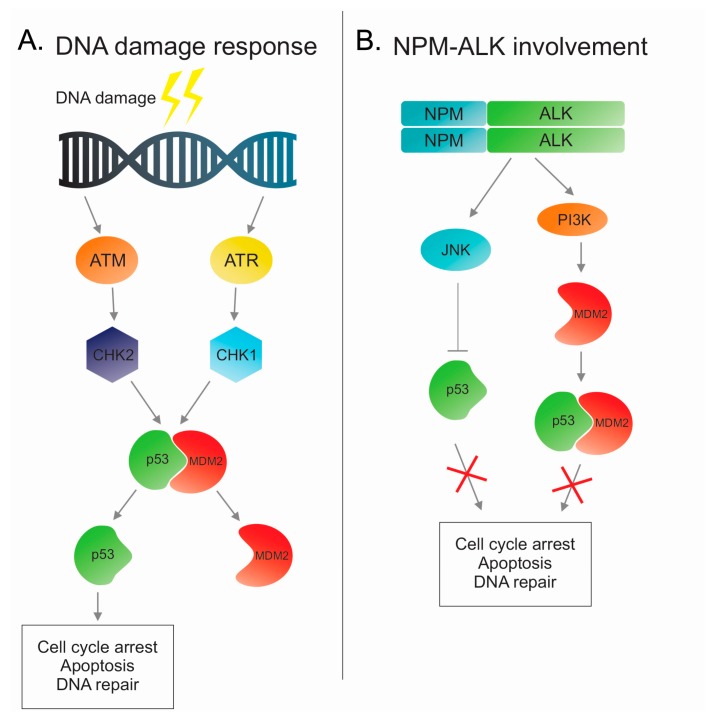
Involvement of nucleophosmin (NPM)-anaplastic lymphoma kinase (ALK) in DNA damage response pathway. (**A**) A schematic overview of the DDR pathway with the stimulation of ataxia telangiectasia mutated (ATM) or ATM-Rad3-related (ATR) after DNA damage and the following cascade including p53 activation. (**B**) NPM-ALK activates (Jun-N-terminal kinase) JNK or phosphatidylinositol-3-kinase (PI3K) pathways and the subsequently inhibits the p53 pathway.

The emerging evidence of the role of p53 role in cancer development has prompted researchers to investigate this transcription factor in various tumors, including anaplastic large cell lymphoma. Early genetic studies showed that p53 is uncommonly mutated in ALCL (<10%) and that it is frequently expressed [40,41]. A more recent study, using high throughput technologies, showed that the loss at 17p13 encompassing *TP53* gene, together with the loss at 6q21, are the most frequent lesions in ALCL [42]. The most common ways that are used by cancer cells to inactivate p53 are by mutating *TP53* gene or over-expressing its negative regulator (MDM2). Usually, ALK-positive ALCL carries wild-type p53 and does not over-express MDM2, suggesting that, in this tumor, p53 activity is controlled in an alternative way. It has been shown that NPM-ALK induces phosphoinositide 3-kinase (PI3K) [13] and Jun N-terminal kinase (JNK) [43] and by interaction with these molecules is capable of regulating p53 activity. The transcription factor p53 needs to be localized in the nucleus to carry out its tumor suppressor function. Recent studies suggest that NPM-ALK translocation disrupts p53 function by sequestering p53 in the cytoplasm and by inducing its degradation through JNK and MDM2 activities [44,45]. In particular, Cui and colleagues [44] demonstrated that PI3K phosphorylates MDM2 on serine 166, increasing its stabilization and this leads to an increment of p53-MDM2 binding. As is known, this binding leads to p53 localization in the cytoplasm, and thus to its inhibition. Moreover, the phosphorylation of JNK by NPM-ALK translocation influences also p53 activity. Indeed, p-JNK sequesters the tumor suppressor p53 and induces its degradation (Figure 1B). Further proof of the importance of p53 in NPM-ALK malignancies comes from the murine embryonic fibroblast (MEFs) cell line deficient for p53 and transfected with NPM-ALK. Indeed, p53 seems to play a role in blocking the proliferation-inducing senescence. Loss of p53 allows for the NPM-ALK cells to bypass the senescence and manifest a tumor phenotype [45].

Since the transcription factor p53 prevents tumor progression [46] restoring its expression can be used to promote tumor regression [47]. The wild-type status of p53 in ALK-expressing ALCL may represent an important ally in the struggle against cancer. Indeed, the re-activation of p53 suggests a potential strategy for ALK-positive ALCL treatment. Nutlin-3a is a small molecule able to target MDM2 and disrupt p53-MDM2 bound [48]. Drakos and colleagues showed that nutlin-3a inhibits the interaction p53-MDM2 leading to cell cycle arrest and apoptosis induced by p53 re-activation in one ALK-positive ALCL in vitro system [49]. Their data suggest that the p53-MDM2 interaction could become a novel potential therapeutic target in ALK-positive ALCL.

## 3. NPM-ALK Induces Cellular Senescence in Anaplastic Large Cell Lymphoma

**Figure 2:**
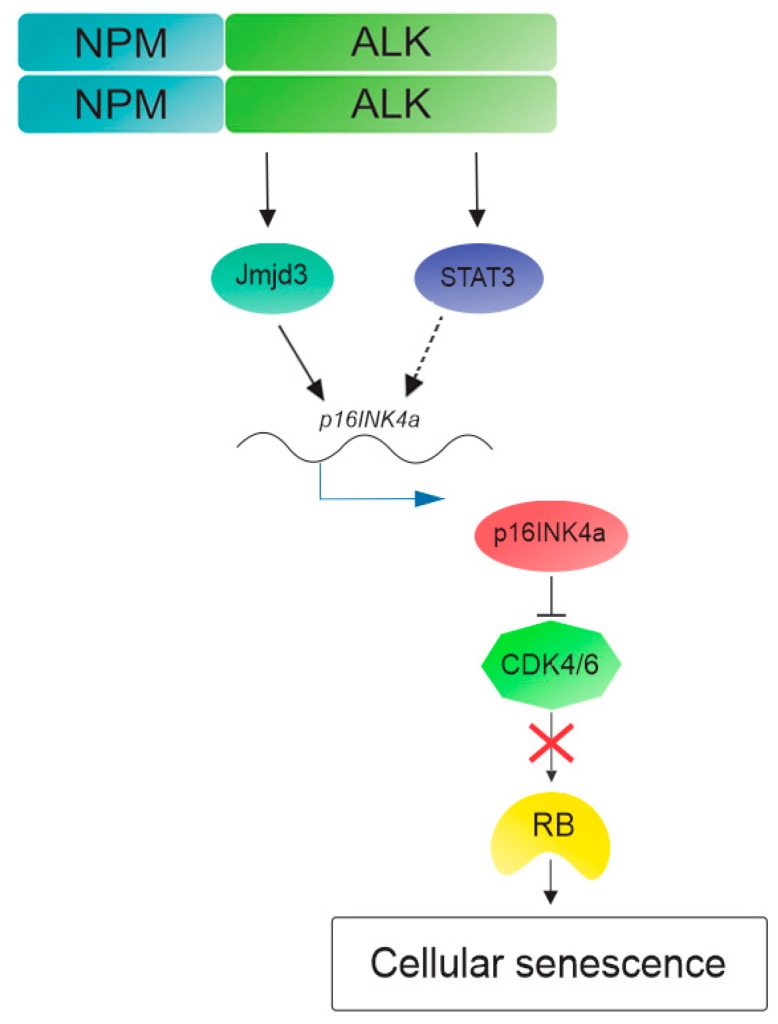
Cellular senescence pathway activated by NPM-ALK. NPM-ALK promotes oncogenic-induced senescence through de-methylation of *p16INK4a* promoter performed by Jmjd3. Multiple mechanisms participate in p16INK4a activation, including signal transducer and activator of transcription 3 (STAT3). Then p16INK4a inhibits cyclin-dependent kinase 4 and 6 (CDK4/CDK6) allowing for retinoblastoma (RB) to induce cellular senescence and cell cycle arrest.

Cellular senescence is a stress response resulting from several mechanisms (e.g., DNA damage, oncogene activation, or telomere shortening) that is aimed at protecting the cell against cancer development or organismal aging [50]. A particular type of cellular senescence mechanism is the oncogene-induced senescence (OIS). In response to DNA damage or various forms of stress induced by expression of activated oncogenes, particularly in the early stage of tumorigenesis, the cell activates cellular senescence pathways inducing irreversible growth arrest [51]. Two of the primary pathways that are involved in OIS mechanisms are p16INK4a/retinoblastoma (RB) and alternative reading frame (ARF)/p53. These proteins trigger the senescence cascade and their high expression can be used as senescence markers, although there are cases in which the overexpression of these markers is not correlated with senescence status, because downstream mutations allow the malignant tumor to overcome the senescence [52]. Oncogenes induce accumulation of DNA damage with consequent activation of ARF and p16INK4a, which respectively stabilize p53 and maintain RB in its active form (hypophosphorylated RB). These two pathways, ARF/p53 and p16INK4a/RB, are frequently mutated in human tumors, and inactivation of these pathways allows the tumor cell to overcome the senescence barrier and induce tumor transformation [53]. Attenuation of ARF and p53 is a typical behavior of several oncogenes in hematological malignancies, such as BCR-ABL in CLL [54], PML-RARα in promyelocytic leukemia [55], and specifically NPM-ALK in ALCL [44]. Transfection of NPM-ALK in primary MEFs shows that cells try to counteract this transformation using cellular senescence mechanisms, in particular p53 and RB pathways [45,53]. As previously described, NPM-ALK inhibits p53 through MDM2 and JNK [44] and ectopic expression of ALK translocation in MEFs shows the deregulation of not only p53, but also the p16INK4a/RB pathway. Indeed, Martinelli et al. [53], showed, in vivo and in vitro, that NPM-ALK induces DNA damage and senescence by activation of p16INK4a, and that consequently, its loss is necessary for ALCL development. Supporting this hypothesis, are the expression data of p16INK4a and RB in ALCL. Low levels or no expression of p16INK4a and expression of inactive RB (phosphorylated RB) were found in NPM-ALK human samples and ALCL cell lines. This study also investigated the mechanisms through NPM-ALK induces the OIS in ALCL, showing that *p16INK4a* is repressed by methylation in its promoter and oncogenic tyrosine kinase induces de-methylation by Jmjd3, a histone lysine demethylase, and then *p16INK4a* transcription. Further data shows that the activation of p16INK4a is due to multiple signals, including Jmjd3 and STAT3, the second one known to be expressed by NPM-ALK [12] (Figure 2). Taken together, these data reveal the ambivalent behavior of NPM-ALK, acting on pathways that are predisposed to arrest the proliferation and cancer development. These cellular senescence pathways are frequently silenced in ALCL and reactivation of their activity, for instance, with specific de-methylating agents, might be a target for a novel therapeutic strategy.

## 4. NPM-ALK Overexpression Lead to DNA Damage Response activation

As we have described so far, NPM-ALK interferes with the DNA damage response pathway, aiming to circumvent cellular senescence and inducing cancer progression. Surprisingly, a recent study reported an excess of NPM-ALK as leading to the activation of ATM/Chk2 pathway, involved in DDR signaling, and promoting apoptosis [56]. NPM-ALK is equally distributed between nucleus and cytoplasm, but it has been shown that only the cytoplasmic portion is active and that the nuclear portion is sequestered in the nucleus together with the wild-type NPM1 [56]. Indeed, the NPM portion in the translocation is lacking in nuclear localization sequence (NLS) and nucleolar localization signal (NuLS), domains that allow wild-type NPM1 to translocate in the nucleus. Overexpression of NPM-ALK, in vitro and in vivo, increases the cytoplasmic portion of the translocation leading to activation of caspase 3 and 7 and apoptosis. As in ALCL, these results showed that the balance of cytoplasmic and nucleus portion of NPM-ALK is crucial. Moreover, the stress that was caused by NPM-ALK overexpression led to the DNA damage response activation, mainly through ATM/Chk2 pathway and H2A histone family member X (H2AX) phosphorylation caused by the increase in MAPK/ERK1/2 activation. Notably, genomic amplification of the *ALK* locus has been described in ALK tyrosine kinase inhibitors (TKI) resistance [57,58]. Indeed, in NPM-ALK cell lines harboring *ALK* amplification showed a higher localization of ALK-translocation in the cytoplasm and TKIs resistance. Interestingly, and necessary for further investigation, is the behavior of NPM-ALK amplified cells treated with TKIs upon drug withdrawal. In this condition, the cells activate the pathways that are previously described in the presence of NPM-ALK overexpression, with high activation of MAPK/ERK1/2, phosphorylation of H2AX and increased cell death [56]. This remarkable behavior of NPM-ALK overexpression after TKIs suspension warrants further investigation and it could represent another valid therapeutic option in TKIs resistance ALK-amplified ALCL using the DNA damage response pathway as an ally.

## 5. DNA Repair

DNA damage can be manifested in a variety of forms; hence, the cell developed a complex DNA repair system specific to different types of DNA lesions. Concerning mammalian cells, one can identify five distinct mechanisms, by which the DNA repair machinery works; base excision repair (BER), DNA mismatch repair (MMR), nucleotide excision repair (NER), DNA strand break repair, including homologous recombination (HR), and nonhomologous end joining (NHEJ) [59,60]. For the aim of this review, we will focus mainly on DNA mismatch repair in which NPM-ALK appears to play a part.

### 5.1. DNA Mismatch Repair

During DNA synthesis, the fidelity with which each copy of DNA is duplicated is critical. Base-base mismatches or insertion/deletion loops (IDLs) can possibly occur, particularly in DNA regions with repeated-sequence motifs. If these errors remain unrepaired, they can evolve in single nucleotide variations (SNVs), modify the phenotype and lead to disease. To prevent these kinds of events during DNA synthesis, and to maintain genomic stability, the cells developed a specific mechanism—DNA mismatch repair (MMR) [61]. The MMR pathway is highly conserved across species and its homologous MMR components that are present in *Escherichia coli* have also been found in the mammalian cell [62,63]. The MMR pathway needs first to recognize the mismatch, then remove the fragment of the newly synthesized strand containing the error and ultimately resynthesize it correctly. In human cells, there are two ATPases protein complexes assigned to recognize mismatches: MuTSα and MuTSβ complexes. The first and most abundant is formed by MSH2 with MSH6 (MutS protein homolog 2 and 6). It preferentially recognizes the base-base mismatches and the small insertion/deletion mispairs. The second one is formed by MSH2 with MSH3 and it is charged to the recognition of larger insertions/deletions [64,65]. Once the mismatches have been recognized, another complex, MutL, links the MuTS, triggering the MMR downstream cascade [66,67]. Then, the proliferating cellular nuclear antigen (PCNA) interacts with MutL/MuTS complex introducing a nick to the mismatch region [68,69]. Exonuclease I (EXO1) catalyzes the excision of the newly synthesized DNA, removing the mismatch and the PCNA stimulates the DNA polymerase δ activation for the re-synthesis of the DNA strand. The remaining nick is then ligated by DNA ligase I and in this way the mismatch is corrected [70,71].

### 5.2. DNA Mismatch Repair in ALK-Positive Anaplastic Large Cell Lymphoma

**Figure 3:**
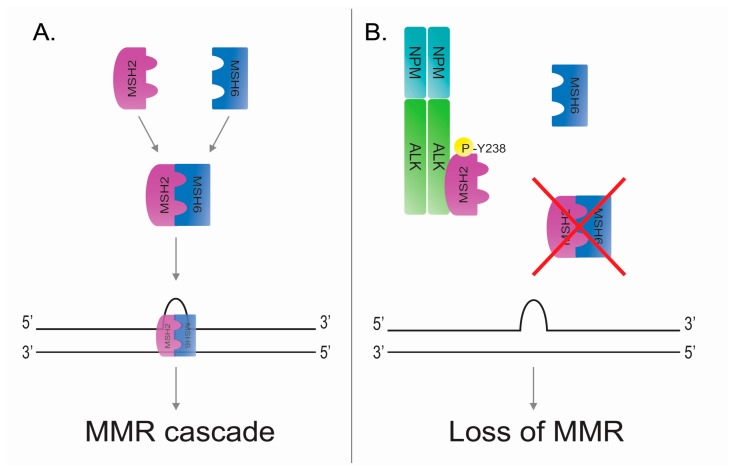
NPM-ALK mediates the phosphorylation of MutS protein homolog 2 (MSH2) at tyrosine 238 leading to loss of DNA mismatch repair (MMR). (**A**) Physiologic activation of MMR after mismatch. MSH2 and MSH6 can interact forming a MuTSα complex and then translocating to the nucleus where the complex actives the MMR cascade. (**B**) Oncogenic tyrosine kinase NPM-ALK phosphorylates MSH2 at tyrosine 238 and it avoids the MSH2:MSH6 interaction and the normal activation of MMR mechanism in the presence of DNA mismatches.

Defects in the MMR mechanism increase the number of spontaneous mutations [72]. Indeed, MMR deficiencies have been associated to different diseases [62], such as hereditary non-polyposis colorectal cancer (Lynch syndrome) [73,74,75], as well as to resistance to chemotherapy drugs or abnormalities in meiosis [76,77]. Moreover, knockout mice deficient for MMR genes (*MSH2*, *MSH6*, *MLH1*, *PMS2*, *EXO1*) showed a high presence of microsatellite instability (MSI) and susceptibility to lymphoma development [77,78,79,80,81]. The fusion tyrosine kinase NPM-ALK also plays a role in the de-regulation of MMR mechanism in anaplastic large cell lymphoma. A proteome-wide study identified a large number of proteins interacting with NPM-ALK, including minichromosome maintenance complex component 6 (MCM6) and MSH2, which are both involved in DNA repair mechanisms [82]. Moreover, a direct interaction between NPM-ALK and MSH2, and the presence of microsatellite instability (MSI) in ALK-positive ALCL patients has been proven [83]. MSH2 is the key protein in the MuTS complexes. NPM-ALK interacts with MSH2, but not its normal partners in MuTS complexes (MSH6 or MSH3) [82]. In fact, subsequently, it has been shown that NPM-ALK phosphorylates MSH2 (at tyrosine 238) blocking the MSH2-MSH6 interaction (Figure 3B). MSH2 phosphorylated at Y238 is unable to bind MSH6, and then to recognize DNA mismatch, inducing loss of MMR activity in the presence of DNA damage [83,84]. The clinical significance of this finding needs further investigations, and restoring MMR mechanism strategy can be employed in the development of novel cancer treatment.

## 6. Conclusions

NPM-ALK clearly plays the main role in ALCL development modifying, not only the proliferation and survival pathways [15], but also acting on the genomic stability. The ALK translocation is able to indirectly regulate tumor suppressor p53 and directly p16INK4a and MSH2, acting in the DNA damage response and DNA repair pathways. Defects in these two mechanisms induce genomic instability and the accumulation of mutations, leading to higher cancer susceptibility. The standard first-line treatment of ALCL is the patient subjection to chemotherapy regimens. Nowadays, tyrosine kinase ALK inhibitors have been developed, such as Crizotinib, Ceritinib or Lorlatinib [85]. Despite the enormous efforts in the last years, a subgroup of patients still relapses and/or develops resistance to the treatment. Therefore, considerable effort needs to be undertaken to investigate new therapeutic strategies, such as nutlin-3a utilization. Targeting and restoring the normal condition of DDR and DNA repair pathways could turn out to be a new therapeutic strategy to fight cancer development. The phase I of several clinical trials using Nutlin-3a have been already completed in hematological neoplasm and solid tumors, including lymphoma (NCT00623870, NCT00559533). RG7112 is a small molecule derives from Nutlins and the first MDM2 inhibitors that are used in clinical trials. The results from a phase I study in patients with hematologic malignancies provide that MDM2 inhibitor has a sufficient clinical activity in restoring p53 function [86]. On the other hand, the trial highlights clinical adversity, such as hematological toxicity. In this regard, a new compound, RG7388, known as idasanutlin [87], is now under clinical studies. It is a second-generation MDM2 inhibitor and it needs a lower concentration to act respect RG7112. Moreover, a combination of MDM2 inhibitors with other agents, such as monoclonal antibodies, was tested with promising preliminary data [88].

As we described in this review, DNA damage response and DNA repair represent a complex machinery involving a wide number of players and an intricate network, therefore, further studies are necessary to investigate the role of other compounds in DNA damage response and DNA repair pathways. Moreover, the evolution of new high throughput techniques, such as next-generation sequencing, allows for investigating a broad spectrum of genes, and in particular, possible mutations that are involved in increasing genomic instability in the cell.
